# First Record of the Mosquito *Aedes koreicus* (Diptera: Culicidae) in Croatia

**DOI:** 10.3390/tropicalmed11100280

**Published:** 2026-09-30

**Authors:** Goran Vignjević, Bruno Grego, Nataša Turić, Nataša Bušić, Renato Zamburlini, Enrih Merdić

**Affiliations:** 1 Department of Biology, University of Osijek, 31000 Osijek, Croatia; goran@biologija.unios.hr (G.V.); ngraovac@biologija.unios.hr (N.B.); enrih@biologija.unios.hr (E.M.); 2 Museo Civico di Storia Naturale di Trieste, Via dei Tominz 4, 34139 Trieste, Italy; bruno.grego254@gmail.com; 3 Department of Agricultural, Food, Environmental and Animal Sciences (DI4A), University of Udine, Via delle Scienze 206, 33100 Udine, Italy; renato.zamburlini@gmail.com

**Keywords:** invasive species, surveillance, community ecology, Korean bush mosquito, population expansion

## Abstract

*Aedes koreicus*, the Korean bush mosquito, has emerged as a significant invasive threat across Europe since its initial detection in Belgium in 2008. Native to East Asia, this cold-tolerant, container-breeding species has established populations in more than a dozen European countries. During entomological field sampling in the Buzet area (Istria, Croatia), two used-tire depots in the village of Štrped were encountered and identified as potentially suitable breeding habitats for container-breeding mosquitoes. Following the initial detection of these mosquitoes’ larvae, the locality was revisited four times from March to May 2026. Dippers of different sizes were used to sample larvae in the tires. Of the 132 larvae collected, eight species were morphologically identified. Nineteen specimens were identified as *Ae. koreicus* and were detected during each visit. The identification of *Ae. koreicus* was confirmed by molecular analysis. Sequencing and analysis of cytochrome c oxidase I showed high similarity among *Ae. koreicus* populations across Europe. This finding confirms the presence of *Ae. koreicus* in Croatia, which could potentially lead to further establishment, and highlights the need for continued surveillance of invasive mosquito species.

## 1. Introduction

Monitoring and surveillance are essential for characterizing dynamic range expansion and understanding the spatiotemporal patterns of invasive species driven by human activities and climate change. Mapping distributional changes, particularly along major transportation corridors, provides insight into how passive transport and increasing climatic suitability facilitate the spread of populations into new areas. More importantly, identifying introduction sites and tracing introduction routes can help develop effective surveillance and control strategies [1].

*Aedes (Hulecoeteomyia) koreicus* (Edwards, 1917) (Diptera: Culicidae), the Korean bush mosquito, originated in eastern Asia and is native to southeastern Russia, China, Japan and the Korean Peninsula [2]. The first occurrence of this species in Europe was recorded in Belgium in 2008 [3], and in subsequent years it was detected in Italy [4] in particular in the neighboring Friuli V.G. region [5], where today it is one of the most frequent species [6] as well in European Russia [7], the Swiss–Italian border region [8], Germany [9], Hungary [10], Slovenia [11], Crimea [12], Austria [13], Kazakhstan [14], the Netherlands [15], the Czech Republic [16], Slovakia [17], and France [18].

According to the latest data from ECDC [18], the species is established in almost all of these countries except the Netherlands, Slovakia, and the Czech Republic, which have only recently reported this invasive species. Given its geographical position at the crossroads of Central and Southeastern Europe, together with intensive international road transport and tourism, Croatia represents a suitable area for the introduction and further spread of invasive *Aedes* mosquitoes.

Since several neighboring countries of Croatia, such as Italy, Slovenia, and Hungary, reported the first findings of *Ae. koreicus* more than 10 years ago, the introduction of this species to Croatia was widely expected [19]. The spread of *Ae. koreicus* in Europe is clearly not as rapid as that of the Asian tiger mosquito *Aedes albopictus* (Skuse, 1894), but recent records in the Netherlands, Slovakia, and the Czech Republic demonstrate its continued expansion. Females of *Ae. koreicus* lay eggs in artificial breeding sites such as containers, similar to *Ae. albopictus* and the Japanese bush mosquito *Aedes japonicus japonicus* (Theobald, 1901). Therefore, its spread could be facilitated by the trade and transport of small containers, ornamental plants, and used tires [3,20,21,22].

Morphologically, adult female *Ae. koreicus* closely resemble *Ae. japonicus* but can be distinguished by the pattern of thoracic lines—specifically, the two secondary lines in *Ae. koreicus* are shorter [18,23]. In addition, the presence of a basal pale band on hind tarsomere V is characteristic of European populations that originated from Jeju-Do Island [24] (Jeju-Do morphotype). This morphotype occurs in all European countries [25] and now also in Croatia, with the exception of one German population [23], which appears to result from a separate, independent long-distance introduction [25,26]. German specimens match those detected in mainland Republic of Korea populations and lack the pale band on hind tarsomere V (mainland morphotype) [27]. Besides their morphological similarity, these species are sympatric both in mainland Korea and Jeju-Do Island [24,28] and can share the same breeding sites [18]. Furthermore, *Ae. koreicus* and *Ae. japonicus* belong to the same monophyletic species complex [28] and can be misidentified if the mainland morphotype is present in the population [27,29,30].

The invasion of *Ae. koreicus* is most likely supported by factors such as adaptable habitat preferences and seasonal occurrence. *Ae. koreicus* produces freeze- and desiccation-resistant dormant eggs that undergo winter diapause, allowing the species to withstand harsh northern European winters and hatch in early spring, which is not the case for *Ae. albopictus* [31]. Additionally, adults show greater persistence during late summer and autumn, and the species becomes active earlier in the season. For these reasons, *Ae. koreicus* appears to be better adapted to colder temperatures, which favors its colonization of hilly and prealpine areas [4,32,33]. Other studies indicate that *Ae. koreicus* is well adapted to urban settlements and can colonize such areas, where various artificial containers serve as breeding sites [33,34]. However, this species is also frequently found in natural habitats, such as forests [23,32], which suggests that *Ae. koreicus* can complete its life cycle in such environments. The species exhibits opportunistic, day-active, and primarily anthropophilic feeding preferences, although it also feeds readily on domestic pets and farm animals such as cattle [18,33,35,36].

Another important aspect of monitoring invasive mosquito species is to “know your enemy,” primarily because these species are often competent vectors of zoonotic and human pathogens. *Ae. koreicus* is already a proven vector of chikungunya virus (CHIKV), Zika virus (ZIKV), and the recently detected Wiesbaden virus (WBDV) [36]. The importance of this species’ vector competence is further heightened by its potential involvement in the transmission of filarial nematodes such as *Dirofilaria immitis* [37], *Dirofilaria repens* [38], and *Brugia malayi* [37,39].

The aim of this research was to detect and confirm the presence of invasive mosquito species in Croatia and to highlight the need for continued surveillance of invasive mosquito species.

## 2. Materials and Methods

### 2.1. Study Area and Mosquito Sampling

**Figure 1:**
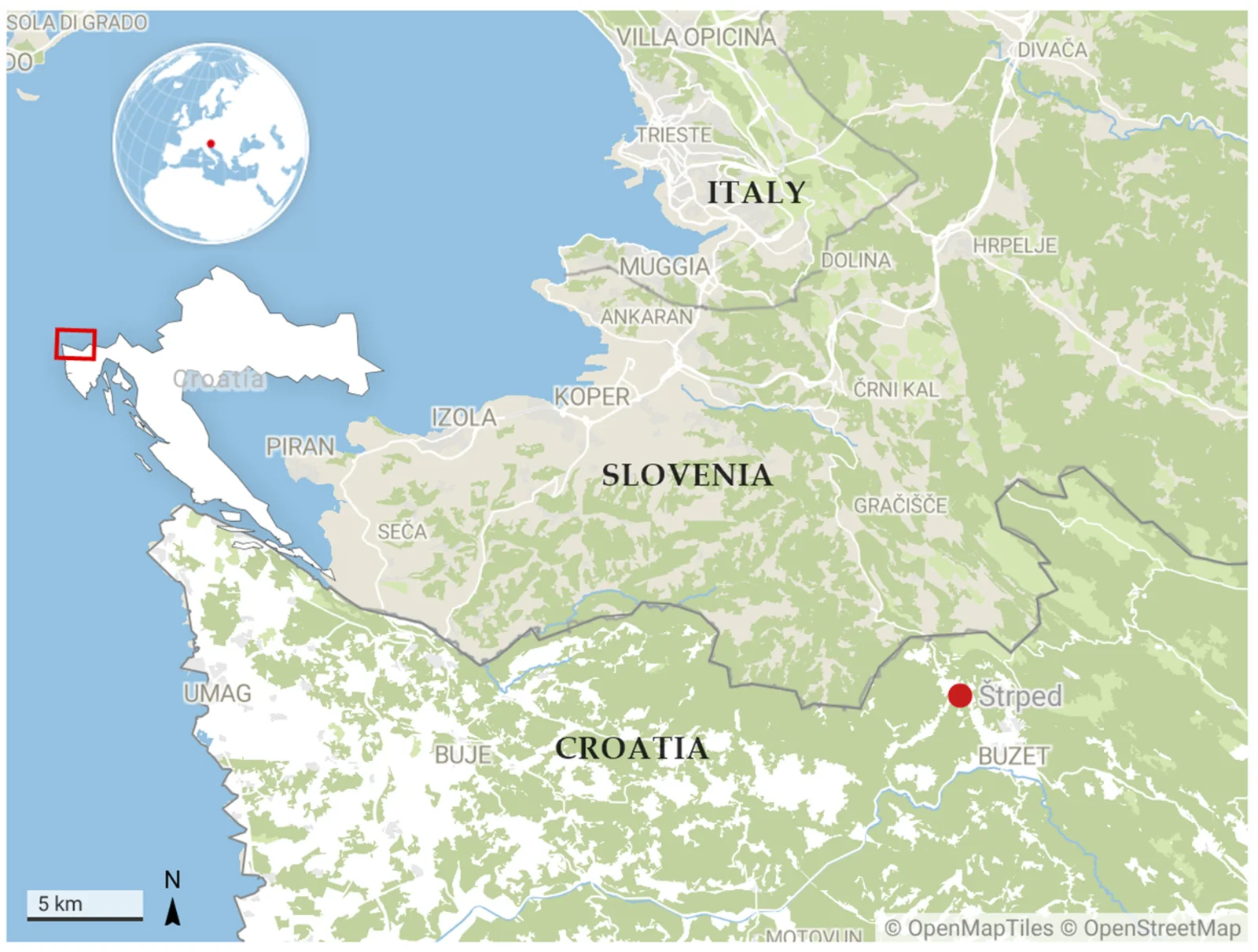
Investigated area. The red dot marks the inspected site (truck tire depot). Map source: ©OpenStreetMap, openstreetmap.org.

During entomological field sampling in the Buzet area (Istria, Croatia), a used-tire depot in the village of Štrped was encountered and identified as a potentially suitable breeding habitat for container-breeding mosquitoes and was therefore inspected for mosquito larvae. The village is located near the Slovenian (4 km) and Italian (30 km) borders along the main road connecting the three countries. The depot is situated on the outer edge of the village (45°25′50.82″ N, 13°56′32.15″ E, 109 m a.s.l.) and contains used truck tires (Figure 1). Larval samples were collected from used tires with a homemade dipper (1 L, 20 cm diameter). The collected larvae and pupae were stored in ethanol (96%) or placed in a plastic mosquito breeder (BioQuip, Compton, CA, USA) and transported alive to the laboratory for hatching. Following the initial detection of *Ae. koreicus*, the locality was revisited to confirm its continued presence. Larval sampling was carried out on 27 March, 18 April, 13 May, and 23 May 2026. During each visit, approximately 20 accessible containers (used truck tires) were systematically inspected for mosquito larvae.

### 2.2. Morphological and Molecular Identification

Larvae and adult mosquitoes were morphologically identified using the keys of Schaffner et al. [40], Becker et al. [41], and ECDC [42]. Temporary water mounts of larvae were prepared to examine comb scales, siphonal pecten, and frontal setae, and slides with dissected male hypopygial structures were made for verification. Specimens were photographed with an Olympus DP25 digital camera (Olympus, Tokyo, Japan) mounted on an Olympus SZX16 stereomicroscope (Olympus, Tokyo, Japan).

Genomic DNA was extracted from previously morphologically identified specimens: one whole larva (sampled on 18 April) and one whole female adult specimen (hatched from a larva sampled on 23 May), using the DNeasy^®^ Blood & Tissue Kit (Qiagen, Hilden, Germany) according to the manufacturer’s protocol. The extracted DNA was stored at 4 °C until further use, and its concentration and purity were measured spectrophotometrically with a NanoPhotometer P330 (Implen, Munich, Germany).

The mitochondrial marker cytochrome c oxidase subunit I (COI) was amplified using the universal primer pair LCO1490/HCO2198 [43]. PCR amplifications were performed in a 25 µL reaction volume with EmeraldAmp^®^ MAX PCR Master Mix (Takara Biotechnology, Kusatsu, Shiga, Japan). For COI, the thermal cycling profile consisted of an initial denaturation at 94 °C for 3 min, followed by 39 cycles of 94 °C for 20 s, 50 °C for 20 s, and 72 °C for 30 s, with a final extension at 72 °C for 5 min. PCR products were visualized on 2% agarose gels, purified, and sequenced bidirectionally by Macrogen Inc. (Amsterdam, The Netherlands). Specimens from the same sampling site are deposited in the Department of Biology, Josip Juraj Strossmayer University of Osijek.

Forward and reverse reads of the partial mitochondrial cytochrome c oxidase subunit I (COI) gene were assembled into consensus sequences and mapped to reference sequences downloaded from GenBank using Geneious Prime 2.2 (Dotmatics, Auckland, New Zealand). Consensus sequences of approximately 1000 bp were aligned with partial COI sequences of *Ae. koreicus* downloaded from GenBank and BOLD using the Clustal Omega algorithm. The newly generated nucleotide sequences were deposited in BOLD under procession IDs CROCU349-26 and CROCU350-26. Phylogenetic relationships were inferred using the Maximum Likelihood (ML) method based on the Hasegawa–Kishino–Yano model with gamma-distributed rates among sites (HKY + G), selected as the best-fitting nucleotide substitution model according to the lowest Bayesian Information Criterion (BIC), implemented in MEGA6 [44,45]. Node support was evaluated with 1000 bootstrap replicates. All positions containing gaps and missing data were excluded, resulting in a final alignment of 658 nucleotide positions from 49 sequences.

## 3. Results

**Figure 2:**
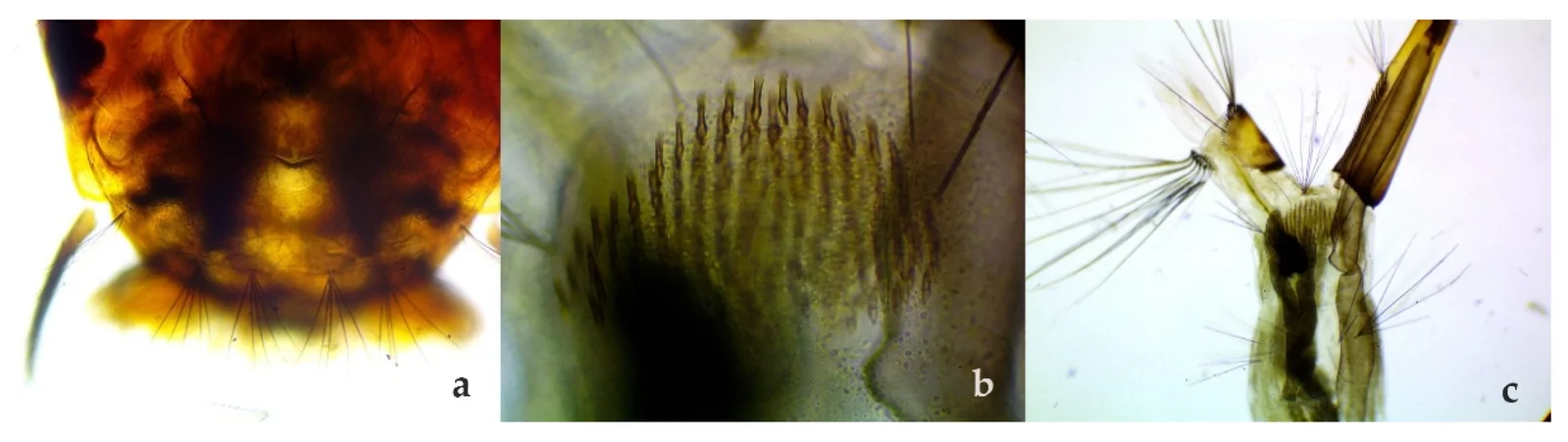
Head (**a**), comb scales (**b**), and terminal abdominal segments (**c**) of *Aedes koreicus* larvae sampled in Štrped, Croatia.

From the samples collected at the truck tire depot and stored in ethanol, we detected larvae of *Ae. koreicus*. The observed characteristics of *Ae. koreicus* larvae included multiply branched frontal setae on the head, comb scales on the abdominal segment without an elongate median spine, and evenly spaced pecten teeth on the siphon that do not extend beyond the insertion point of the siphonal tuft (Figure 2).

**Figure 3:**
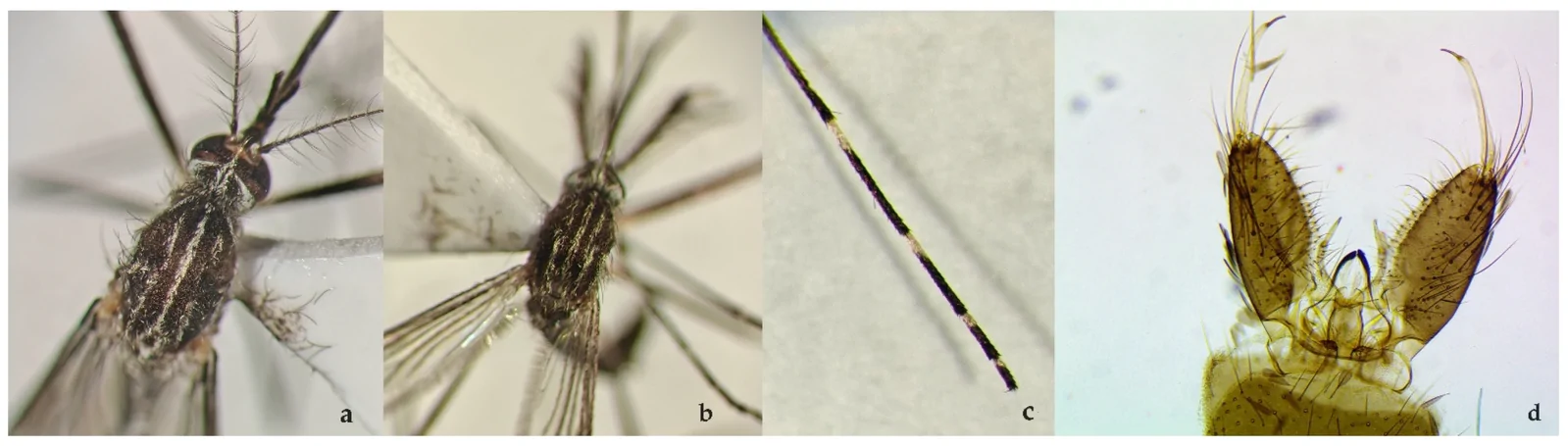
Scutum of the female (**a**) and male (**b**), hind tarsomeres IV and V with pale bands (**c**), and hypopygium of the adult male (**d**) of *Aedes koreicus* reared from the larvae sampled in Štrped, Croatia.

From larvae reared under laboratory conditions at room temperature, we confirmed the presence of *Ae. koreicus* adults based on scutum ornamentation, the basal pale band on hind tarsomeres IV and V, and the shape of the male genitalia (Figure 3).

Including *Ae. koreicus*, eight mosquito species were identified during the survey: *Ae. j. japonicus*, *Ae. albopictus*, *Anopheles plumbeus* (Stephens, 1828), *Culex pipiens* (L., 1758), *Cx. hortensis* (Ficalbi, 1899), *Culiseta annulata* (Schrank, 1776), and *Aedes geniculatus* (Olivier, 1791). Larvae of *Ae. j. japonicus* were the most numerous, followed by *Ae. koreicus*. Both species were consistently present in the inspected tires during each visit. The numbers of collected larvae for each species are presented in Table 1, and the positive detections of the species throughout the sampling period are shown in Table 2.

Two of the collected *Ae. koreicus* specimens (one larva and one adult female) were submitted for molecular analysis, which confirmed the morphological identification. According to the BOLD database, our sequences showed 100% identity with 11 sequences from Italy and the Netherlands, while GenBank analysis revealed the highest similarity (99.8%) with 25 sequences from Italy, the Netherlands, Belgium, Hungary, and Slovakia. Among the BOLD BINs assigned to *Ae. koreicus* specimens, our sequences belonged to BOLD: ACB6413, which contains the largest number of sequences from Europe.

**Figure 4:**
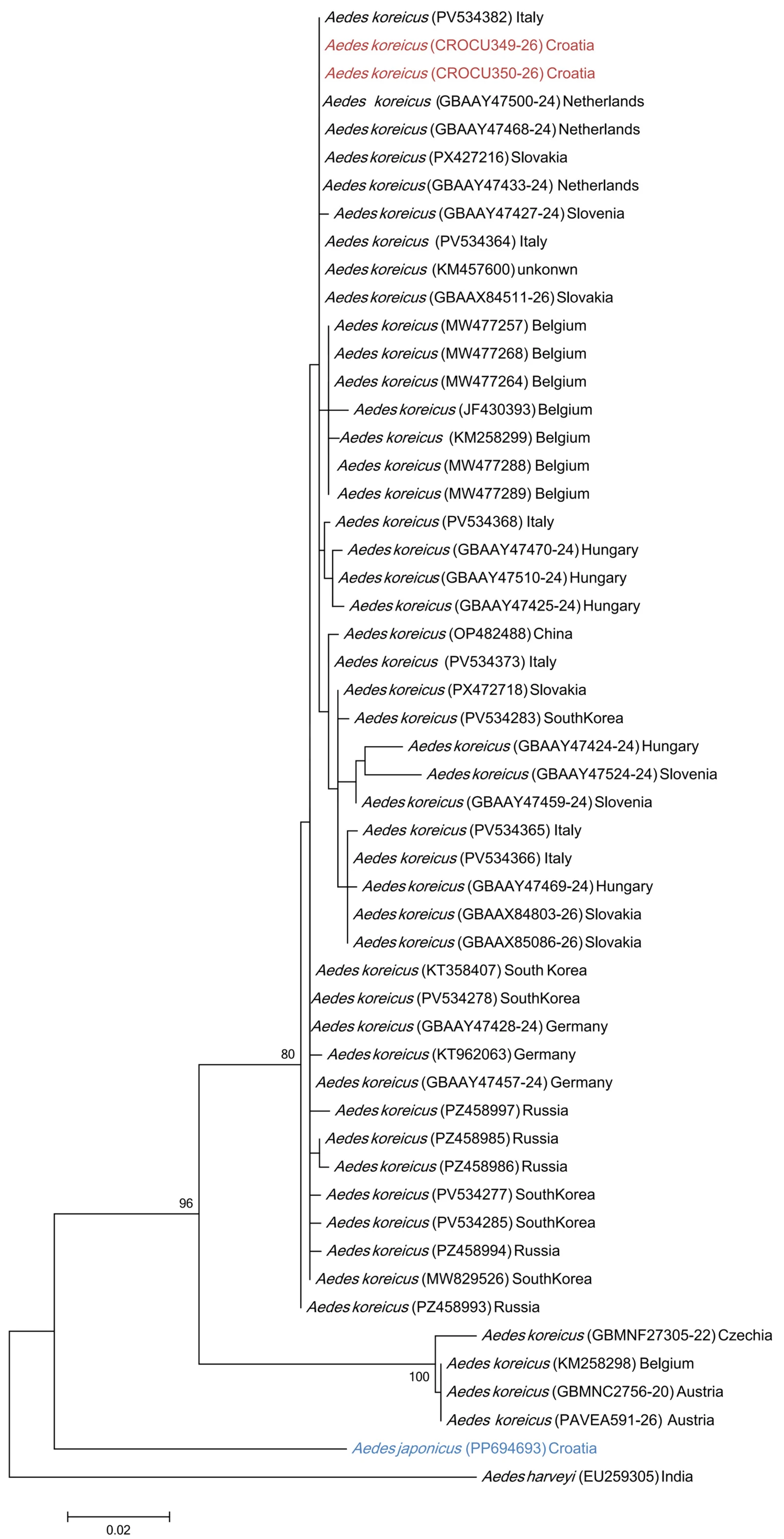
Maximum likelihood phylogenetic tree of partial mitochondrial COI sequences of *Aedes koreicus* (red) and *Aedes japonicus* (blue) isolates from Croatia in comparison with 49 reference sequences of *Aedes koreicus* retrieved from GenBank and BOLD. *Aedes harveyi* sequence was used as the outgroup. Bootstrap support values ≥ 75% are shown above the branches. Branch lengths represent the number of substitutions per site. The sequence labeled with unknown origin (most likely from The Netherlands) is included due to its 100% identity with the Croatian sequence.

Phylogenetic analysis of partial mitochondrial COI sequences resulted in a final alignment of 51 sequences, including the two newly generated Croatian sequences and 49 reference sequences of *Ae. koreicus* retrieved from GenBank and BOLD (Supplementary Table S1). The results showed that all analyzed *Ae. koreicus* specimens clustered together and were clearly separated from the outgroup taxon, *Ae. harveyi*. Given the high morphological similarity between *Ae. japonicus* and *Ae. koreicus*, a Croatian sequence of *Ae. japonicus* was included for comparison and formed a clearly distinct lineage from the *Ae. koreicus* sequences. The two Croatian specimens were positioned within the main *Ae. koreicus* clade and did not form a distinct lineage, clustering among reference sequences from other European countries. Bootstrap support for internal relationships varied considerably, with several nodes showing low to moderate support, indicating limited resolution among closely related COI haplotypes. Nevertheless, several more distinct subclades were recovered, including a cluster comprising specimens from Czechia, Belgium, and Austria. Overall, no clear geographic clustering was observed, as sequences from different countries were largely intermingled throughout the tree (Figure 4). The comparison taxon (*Aedes japonicus* from Croatia) and the outgroup (*Aedes harveyi*) were used exclusively for phylogenetic tree reconstruction, whereas sequences of *Ae. koreicus*, both generated and retrieved, were used for pairwise *p*-distance analysis.

Pairwise *p*-distance analysis of 51 *Ae. koreicus* COI sequences (1275 pairwise comparisons) revealed generally low genetic divergence within the main phylogenetic cluster, whereas a smaller group of sequences showed substantially higher divergence. Overall, *p*-distances ranged from 0.0000 to 0.0713 (mean 0.0123; median 0.0046). The two Croatian specimens showed no sequence divergence over the overlapping COI region (*p*-distance 0.0000) and were also identical to several reference sequences from other European countries. The highest *p*-distance (0.0713; 7.13%) was observed between the Czech specimen GBMNF27305-22 and specimens from Hungary (GBAAY47424-24) and Slovenia (GBAAY47524-24). These elevated distances were associated with the distinct Czechia–Austria–Belgium cluster observed in the ML tree, rather than representing an isolated pairwise comparison (Table 3).

## 4. Discussion

The current Croatian mosquito fauna comprises 52 species [46], including two invasive species, *Ae. albopictus*, recorded in 2004 [47], and *Ae. j. japonicus*, recorded in 2013 [48]. In addition to the detection of new invasive species, the finding of *Ae. koreicus* in Croatia provides new data on the species’ known range in Europe. Moreover, repeated detection at the same locality over a two-month period could potentially lead to further establishment.

Originally native to East Asia, the species has, since it was first recorded in Europe in 2008, become a highly successful invasive species and is now widely established across temperate, pre-alpine, and urban areas of Central and Southern Europe. Driven by passive, human-mediated vehicular transport along major trans-Alpine motorways, its cold-tolerant biology has allowed it to colonize regions where cooler temperatures limit the establishment of other invasive species, such as *Ae. albopictus* [31]. Its arrival in Croatia likely followed the same pattern, as it was first detected along the main road from Slovenia and Italy. According to climate suitability models using the SSP126 scenario, under the influence of global warming and increasing international trade, suitable habitats for *Ae. koreicus* in Europe will expand significantly northward, with high-suitability zones remaining concentrated in northern Italy and southern Germany near the Alps [49].

In invaded territories, *Ae. koreicus* larvae are frequently found in artificial containers such as discarded tires, buckets, flower pots in cemeteries, and road catch basins, but they also exploit natural microhabitats such as tree holes and rock cavities [23,29,50]. It overwinters in the egg stage, using desiccation-resistant eggs that remain in diapause until environmental conditions become favorable in spring [51,52]. The occurrence of larvae in March in this study is consistent with this pattern: no tiger mosquito specimens were present, temperatures were lower, and larvae were found at a used tire depot. We also confirmed that *Ae. koreicus* often shares breeding niches with *Ae. japonicus*, as both species were found at the same breeding sites, even though *Ae. koreicus* appears more frequently associated with human-inhabited areas [23,29]. The co-occurrence of invasive species we observed matches the typical container-breeding profile described in studies from Italy, Hungary, and Germany [4,10,23]. Furthermore, species composition almost perfectly matches documented mosquito communities from similar studies [23], with *Ae. j. japonicus* being the dominant species in larval habitats where *Ae. koreicus* was found [50].

The phylogenetic tree and parsimony distances of the compared COI sequences show that most populations within Europe are genetically closely related, with *p*-distances ranging from 0.00 to 0.07 and with no clear geographic clustering observed. This finding supports a scenario of stable expansion and colonization of neighboring countries rather than multiple introductions [25]. However, the same analyses identified a distinct, highly divergent mitochondrial lineage within the *Ae. koreicus* clade (comprising sequences KM258298, GBMNC2756-20, GBMNF27305-22, and PAVEA591-26), exhibiting pairwise COI *p*-distances of approximately 5.0% to 7.13% relative to the main European lineage. Such intraspecific COI divergence exceeding 7% is well documented in populations and complex species groups within *Aedes* that significantly exceed baselines of 2% to 3% genetic distance [17,53,54,55]. Furthermore, *Ae. koreicus* is known to harbor substantial mitochondrial COI diversity, with previous studies reporting at least 31 distinct haplotypes structured across multiple major haplotype clusters [27]. We therefore interpret this divergent lineage as an established, highly divergent mitochondrial haplotype cluster of *Ae. koreicus* in Europe.

The ability to control *Ae. koreicus* populations is important, and their potential establishment in Croatia is a public and veterinary health concern due to their status as a disease vector. This species is a highly efficient vector for the dog heartworm *Dirofilaria immitis* and the subcutaneous parasite *Dirofilaria repens*, posing a direct risk to domestic and wild mammals [25,37,56]. Although the current risk of human arbovirus transmission (CHIKV and ZIKV) is low under typical European temperature regimes, the discovery of Wiesbaden virus (WBDV) and its synergistic boosting effect on CHIKV replication highlights the potential for local microviromes to alter vector competence and increase disease transmission dynamics [36]. Moreover, its proven vector capacity for Japanese encephalitis virus (JEV) in its native range is concerning, particularly if the species continues to expand into European agricultural or wetland areas containing JEV-amplifying hosts, such as pigs and ardeid birds [18,33,57,58].

To address the spread of this potential vector, public health authorities should prioritize standardized surveillance along major transit axes, integrate genetic diagnostics to prevent misidentification with *Ae. japonicus*, and implement targeted, community-level vector control programs in high-risk urban and peri-urban habitats [8,27,50,59,60].

## Figures and Tables

**Table 1:** Overall cumulative larval abundance by species.

Species	Number of Collected Larvae
*Ae. j. japonicus*	76
*Ae. koreicus*	19
*Ae. albopictus*	16
*Cx. pipiens*	7
*Cx. hortensis*	5
*Ae. geniculatus*	4
*Cs. annulata*	3
*An. plumbeus*	2

**Table 2:** Occurrence of collected species in larval samples during the sampling period. “X” represents presence, while “-” represents absence of at least one positive container.

Species	27 March	18 April	13 May	23 May
*Ae. j. japonicus*	X	X	X	X
*Ae. koreicus*	X	X	X	X
*Cx. pipiens*	X	X	X	X
*Ae. albopictus*	-	X	X	X
*An. plumbeus*	-	X	-	X
*Cx. hortensis*	-	-	-	X
*Ae. geniculatus*	-	X	-	-
*Cs. annulata*	-	X	-	-

**Table 3:** Summary of pairwise *p*-distances among *Aedes koreicus* COI sequences included in the analysis.

Parameter	Value
Number of sequences	51
Number of pairwise comparisons	1.275
Mean *p*-distance	0.0123
Median *p*-distance	0.0046
Minimum *p*-distance	0.0000
Maximum *p*-distance	0.0713

## Data Availability

In this study, two new COI sequences of *Ae. koreicus* detected in Croatia can be found in the BOLD database (project CROKOR) under procession IDs CROCU349-26 and CROCU350-26 and in GenBank under accession numbers PZ899402 and PZ899403. Other data presented in this study are available from the corresponding author upon reasonable request.

## Supplementary Materials

The following supporting information can be downloaded at https://www.mdpi.com/article/10.3390/tropicalmed11100280/s1, Table S1: Mitochondrial COI Sequence Dataset.
